# Structural and Biochemical Features of Human Serum Albumin Essential for Eukaryotic Cell Culture

**DOI:** 10.3390/ijms22168411

**Published:** 2021-08-05

**Authors:** Vibhor Mishra, Richard J. Heath

**Affiliations:** Protein Production Facility, Shared Resource Center, St. Jude Children’s Research Hospital, Memphis, TN 38105, USA; Richard.Heath@stjude.org

**Keywords:** human serum albumin, cell culture, ligand binding

## Abstract

Serum albumin physically interacts with fatty acids, small molecules, metal ions, and several other proteins. Binding with a plethora of bioactive substances makes it a critical transport molecule. Albumin also scavenges the reactive oxygen species that are harmful to cell survival. These properties make albumin an excellent choice to promote cell growth and maintain a variety of eukaryotic cells under in vitro culture environment. Furthermore, purified recombinant human serum albumin is mostly free from impurities and modifications, providing a perfect choice as an additive in cell and tissue culture media while avoiding any regulatory constraints. This review discusses key features of human serum albumin implicated in cell growth and survival under in vitro conditions.

## 1. Introduction

Albumins are globular proteins commonly found in blood plasma, egg white, milk, and plants [1,2,3,4]. Serum albumin is the most abundant protein in the blood plasma of all vertebrates [5]. It is synthesized in the liver as pre-pro-albumin and matures in the endoplasmic reticulum and the Golgi bodies before being secreted from the hepatocytes [5,6]. Human serum albumin (HSA) has a plasma concentration of 35–50 mg/mL [6,7], an approximate half-life of 19 days, and it is present in both extravascular and intravascular spaces [7,8]. Albumin performs a variety of essential functions. It regulates the oncotic pressure and pH of the blood [5]. It also binds and transports various bioactive molecules, including proteins, peptides, fatty acids, hormones, amino acids, drugs, nutrients, and metal ions [6,9]. These properties make albumin an excellent candidate for several clinical and biotechnological applications.

HSA is clinically used in hemorrhagic shock due to excessive blood loss, hypovolemia, hypoproteinemia, and fetal erythroblastosis [6,10]. In addition, purified HSA is commonly used in eukaryotic cell culture practices [11,12]. The past decade has seen HSA been extensively explored as a nanoparticle for targeted drug delivery [13]. For all these applications, large quantities of HSA are classically sourced from blood serum. However, recombinant HSA from heterologous sources such as *Pichia pastoris*, *Saccharomyces cerevisiae, Escherichia coli*, *Kluyveromyces lactis*, transgenic animals, and plants have proven to be most beneficial for biotechnological purposes [14,15,16,17,18,19,20,21,22].

Albumins are extensively used as drug delivery vehicles for various ailments due to their high serum concentration, long half-life, frequent recirculation, abundant accumulation in benign and malignant tissue types, non-toxicity, and non-immunogenicity [8,23,24]. Albumin quickly diffuses across leaky blood vessels in tumors, making it ideal for carrying anticancer drugs [24]. A large and diverse variety of drug molecules can be very effectively bound to the albumin-based nanoparticles (NP) [13,25]. These albumin NPs have been shown to have high drug entrapment capacity, controlled release, and high biocompatibility, and they are also biodegradable [13,25]. Moreover, albumin NPs are designed for efficient drug loading [26]. The NP can also accommodate surface modifications for improved drug binding, solubility, and enhanced drug targeting in a controlled fashion [13]. The conjugation of drugs to the albumin can be achieved by covalently linking the therapeutic compound to the N- or C-terminus of the protein or any other unique amino acid of albumin using click chemistry, recombinant DNA technology, chemical cross-linking, or non-covalent interaction [24,27,28]. 

Traditionally, fetal bovine serum (FBS) was a critical factor in eukaryotic cell cultures. It provides essential elements required for the desired growth of cells under in vitro conditions [11]. A crucial factor in FBS is bovine serum albumin (BSA), which accounts for >95% of the protein content of the serum, along with small amounts of other proteins, including insulin, hormones, and growth factors. However, being a biological product, FBS exhibits significant batch-to-batch variability. It is often found to be contaminated by pathogens such as mycoplasmas, viruses, and prions responsible for transmissible spongiform encephalopathies (TSE). Therefore, there is a desire to avoid the use of serum, especially among those growing cells in a Good Manufacturing Practice (GMP) environment. Nowadays, purified recombinant albumin from heterologous sources has replaced serum in the cell culture media due to increasing regulatory concerns and quality control [29,30]. In addition, the structural similarities between albumins from different vertebrate sources allow them to be swapped in cell cultures to attain comparable results [11]. Consequently, bovine serum albumin (BSA) is more frequently used than HSA as its production is more cost-effective [31]. 

The action of albumin in cell culture is primarily dependent on its antioxidant properties, toxin sequestering properties, and transportation of bioactive ligands. This review highlights the inherent structural and biochemical features that allow human serum albumin (HSA) to be exploited for cell culture applications.

## 2. Recombinant HSA

Classically, HSA was commercially produced by fractionating human plasma [32]. However, human plasma always has a limited supply. In addition, inconsistencies in the quality of the raw material from different sources and other contamination issues lead to variations in the quality and quantity of the final purified protein.

Recombinant DNA technology has played a crucial role in the large-scale production of high-quality recombinant HSA (rHSA) [12]. Yeast, in particular, *Pichia pastoris*, is the most promising source for rHSA production, with easy scale-up of the cell culture to 5000–7500 L [15,33,34,35,36]. Yeast cells provide several post-translational modifications such as proteolytic processing, folding, and disulfide bond formation and can be genetically manipulated to avoid undesirable post-translational modifications [6]. Secretion of the protein into culture media lowers the number of downstream purification steps [15]. A series of ion exchange and hydrophobic chromatographic resins are used to clean up the recombinant albumin to attain desired purity [15,37]. 

Plant seed bioreactors are another promising method for recombinant albumin production. Recombinant HSA can be produced in *Oryza sativa* seeds [14,38,39]. The expression level of the rHSA can be at least 0.3% based on the rice grain’s weight. rHSA produced in this manner is structurally and functionally equivalent to plasma-derived HSA [14]. rHSA is also stably produced to 0.7% of total soluble protein in transgenic tobacco cell suspension culture [16]. Similar to yeast, in this approach, the recombinant protein is secreted into the cell culture media. The media is further subjected to downstream chromatographic processes to achieve desired purity [16].

Heterologous prokaryotic expression hosts have been used for rHSA expression and purification, with only moderate success due to the large size and many required disulfide bonds. Suboptimal processing of the recombinant albumin peptide due to lack of the eukaryotic protein folding machinery results in misfolded protein. Minimal secretion of the recombinant albumin is achieved from *Bacillus subtilis* as higher levels of expression overwhelm the secretion pathway [40]. In *Escherichia coli*, rHSA tends to accumulate as unfolded, insoluble aggregates in inclusion bodies, requiring denaturation and refolding to obtain a suitably active product [19]. These factors are major bottlenecks that increase the number of purification steps, production cost, and highly stringent quality control to achieve the desired quality and quantity of recombinant HSA. Co-expression of rHSA in *E. coli* along with the chaperone proteins can increase the amount of rHSA expressed in the soluble fraction from 10% to 60% [18] and this protein appears to be monomeric and structurally similar to HSA purified from plasma [41]. Some success has also been achieved by engineering HSA as a fusion protein with maltose-binding protein (MBP). Co-expression with protein disulfide isomerase enhances the recombinant protein’s solubility [19].

## 3. Structural and Biochemical Features of HSA

HSA is encoded by a single gene mapped on the long arm of chromosome 4 at position q13.3 [7]. Hepatocytes synthesize albumin as pre-pro-albumin [42]. This pre-mature form of the protein has a 24 amino acid N-terminal extension [43]. These 24 N-terminal amino acids facilitate transport into the endoplasmic reticulum. Once inside, the first 18 amino acids are cleaved to make pro-albumin [43]. This pro-albumin is the primary intracellular form of albumin before it gets transported into the Golgi apparatus. The remaining six amino acids are cleaved from the N-terminus by furin, and the mature albumin is secreted from the hepatocytes [43]. The mature human albumin consists of 585 amino acids and has a molecular mass of 66,348 Da. 

**Figure 1:**
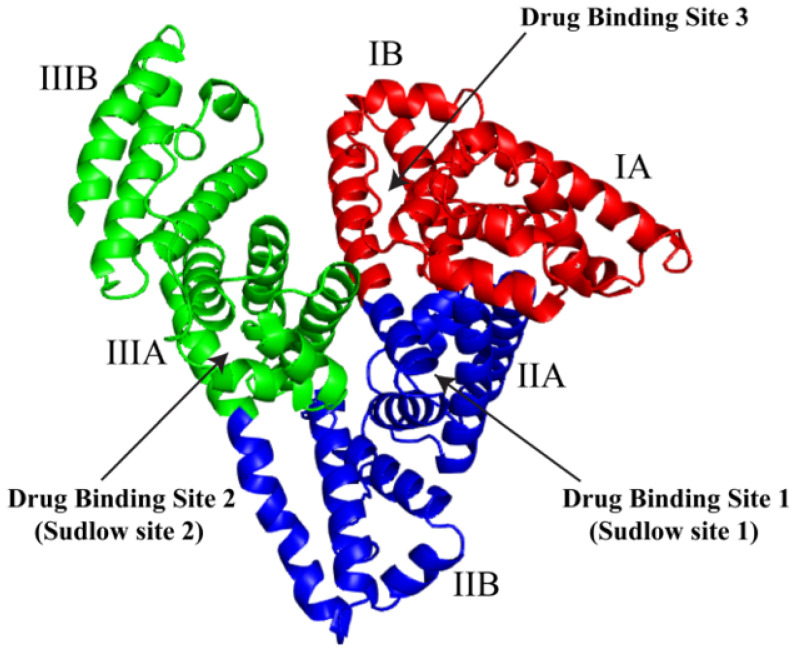
Domain organization of human serum albumin. Domain I (red), domain II (blue), and domain III (green). The 3-D model was generated by PyMOL using 1AO6 PDB file.

**Figure 2:**
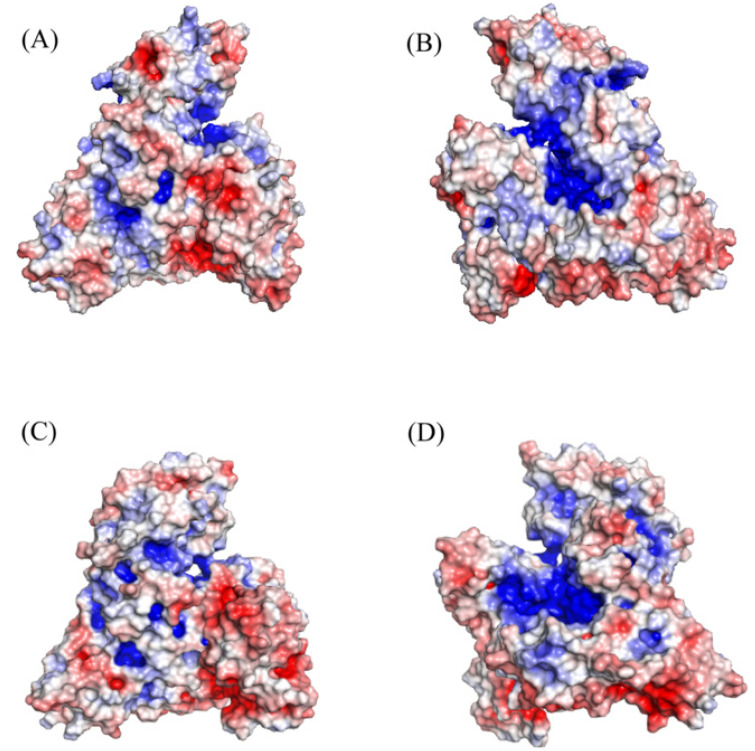
A comparison of surface charge distribution between HSA (**A**,**B**) and BSA (**C**,**D**). The two views for each protein structure are flipped 180° along the vertical axis. The images were generated by PyMOL using 1AO6 and 4F5S PDB files.

HSA is a globular, heart-shaped protein with a repeating series of six helical subdomains [44,45]. HSA is comprised of 67% α-helices, 10% turns, 23% random coils, and no β-sheets [46]. High-resolution X-ray crystallography structures show three predominant domains in albumin. They are generally numbered as domain I (1–195 aa), domain II (196–383 aa), and domain III (384–585 aa) [44] (Figure 1). Each domain is subdivided into two subdomains, A and B. There is a remarkable degree of sequence and structural similarity as well as surface charge distribution similarity between HSA and its counterparts from bovine (BSA), equine (ESA), leporine (LSA), and canine (CSA) sources (Figure 2) [47,48,49]. At a sequence level, HSA and BSA share 76% identity [50], while, overall, serum albumins from various sources share more than 62% sequence identity [48]. The average root mean square deviation (RMSD) of BSA crystal structure compared to HSA structure is 1.1 Å; for ESA and LSA, it is 1.2 Å [48]. This structural similarity is one of the main reasons HSA can be substituted with BSA or albumins from other sources in cell culture practices [11]. Molecular dynamics analysis of HSA, BSA, and CSA indicate that motion of domains I and III are key in defining the properties of the albumins [49]. BSA is structurally somewhat more rigid than HSA, while CSA is more flexible and possesses larger, more water-accessible drug binding sites.

HSA has 17 intramolecular disulfide bridges that are present primarily between the α-helices. These disulfide bonds are essential for the stability of the protein [51]. It also has one free cysteine residue (Cys-34), present in domain I and conserved across species. This residue is responsible for albumin dimerization during purification by forming an intermolecular disulfide bridge [44]. Chemical modification of Cys-34 can prevent dimer formation [52]. Purification of only domain I also results in protein dimerization because of this free cysteine residue [53]. The free cysteine residue is in a ≈10 Å deep crevice and plays a critical role in the redox properties of albumins shown to be crucial in cell culture [11]. Cys-34 forms complexes with various metal ions and scavenges free radicals under in vivo and in vitro conditions [11]. The Cys-34 residue is also involved in scavenging the free radical nitric oxide and other reactive oxygen species (ROS) [54]. It is attributed to protection against lipid peroxidation by reactive oxygen species by scavenging ROS [55]. Additionally, a conserved histidine residue (His-3) also acts as a critical metal chelator that scavenges reactive oxygen species [56].

## 4. Fatty Acid Interactions

HSA binds and carries fatty acids in the vascular system [57]. Albumin is the carrier of 99% of non-esterified fatty acid (FA) present in blood plasma. Classically, albumin is shown to have 7 high- and more than 20 low-affinity FA-binding sites [6,58]. FA affinity to albumin is also chain length-dependent, with oleate (16 carbons) having a higher affinity than laurate (10 carbons) [59]. FA binding modulates conformational changes in albumin [60]. Defatted albumin is present in a specific conformation know as N-form (neutral-form) [61]. The FA bound form of albumin is known as B-form (basic-form) [58,61]. These forms have also been observed as FA-free HSA undergoes reversible conformational transitions at different pH values [62,63]. At pH of lower than 3, HSA has an extended conformation [63,64]. Between pH 3 to 4.3, HSA assumes a fast migrating (F) form characterized by increased viscosity and lower solubility [63]. Between pH 4.3 and 8.0, the N-form is represented by the characteristic heart-shaped structure (Figure 1) [63]. At a pH greater than 8.0, HSA is present as the B-form [63]. The transition from N to B forms is characterized by domain rotation within the molecule; domains I and III in particular appear to pivot around a point close to the interface with domain II [58]. The most distal subdomains display the most significant deviations in position as a consequence of FA binding [58].

**Figure 3:**
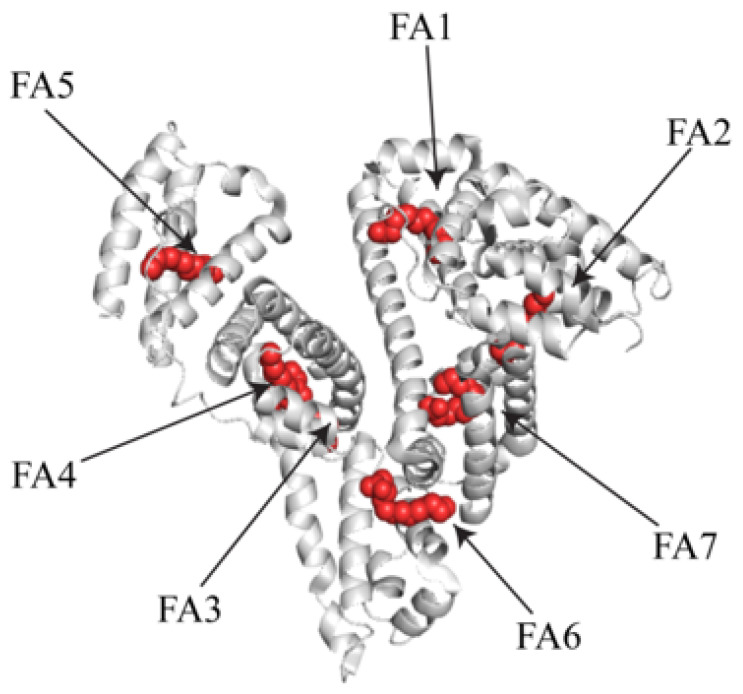
3-D model of HSA showing the seven fatty acid (FA) binding sites. Myristate occupying the seven sites is rendered in red spheres. The 3-D model was generated by PyMOL using 1HK4 PDB file.

The seven high-affinity FA binding sites are asymmetrically distributed throughout the protein [65,66,67] (Figure 3). The first site is located in subdomain IB. This FA binding site also binds with heme, sequestering free heme in the blood and subsequently recirculating it. The second FA-binding site lies at the interface between IA and IIA. FA-binding sites three and four are present in subdomain IIIA. These FA bind sites are also therapeutic compound binding hotspots [68]. The FA-binding site five is present in IIIB. Site six is between subdomains IIA and IIB, and site seven is present in subdomain IIA. NMR studies show sites two, four, and five are the primary FA interacting sites. They offer the most favorable conditions by providing a highly enclosed environment that allows the aliphatic chain of the FA to be tightly bound. Additionally, the presence of basic amino acid side chains at the binding pockets edge leads to salt bridge interactions with the fatty acyl carboxylic head group [58].

FA binding sites also accommodate other ligands, including several drug molecules [58]. Albumins from different species, such as human, bovine, and equine, are structurally similar, with similar amino acid compositions within the FA binding sites [47]. In eukaryotic cell culture systems, albumins present in the media bind with the FAs, circulate them, and help facilitate the FA uptake by the cells [6].

## 5. Metal Ion Interactions

Metal ions are essential for the growth and development of cells [69]. HSA is a key transporter of the crucial metal ions Cu^2+^ and Zn^2+^ in plasma [70]. In addition, metal ions such as copper undergo univalent redox reactions that catalyze the formation of free radicals [71]. It has been shown both in vivo and in vitro conditions that the potential toxic activity of metal ions is mitigated by albumin binding [6]. Albumin has four metal-binding sites with partially selective metal affinity preferences [6,72].

### 5.1. The N-Terminus Metal-Binding Site (NTS)

The first metal-binding site comprises the first three residues (Asp-Ala-His in HSA) at the N-terminus of the protein [6,72,73]. The nitrogen atoms of the peptide bonds between these residues, the N-terminal amine and His-3 residue, coordinate with the Cu^2+^ and Ni^2+^ metal ions in a square planar ligand arrangement [6,73,74]. Cu^2+^ binds with high affinity to albumin, with the dissociation constant for the HSA-Cu^2+^ complex being 6.7 × 10^−17^ M [75]. The His-3 residue is considered critical for the high-affinity binding of Cu^2+^. His-3 is highly conserved in mammals, except dogs and pig albumins [75]. They have His-3 substituted with Tyr-3, leading to higher suscpetibility of copper toxicity in these species [72]. The Ni^2+^ affinity to the NTS is comparatively lower, with a dissociation constant value of 2.5 × 10^−10^ M. Co^2+^ ions also bind to the NTS and have a dissociation constant of 1 × 10^−4^ M [74]. The NTS has a highly flexible conformation as it is not observed even in the high-resolution crystal structures of albumin. 

### 5.2. The Cys-34 Metal-Binding Site

The second metal-binding site is present at reduced Cys-34 residue [6,72]. It is the only cysteine residue within HSA that does not pair with another cysteine to form an intramolecular disulfide bond [45]. The disulfide bond patterns are highly conserved in all vertebrate albumins with 17 disulfide bridges and a single free thiol cysteine residue [45,47,76]. HSA, in its reduced form, has the free thiol of Cys-34. However, this amino acid residue tends to form a heterogenic disulfide bond with other cysteines, leading to protein dimerization [44]. Cys-34 is located in a cleft between helices 2 and 3 of subdomain IA, which results in limited accessibility and high specificity in metal ion interactions [44,72]. Ag^+^, Au ^+^, Hg^2+^, Pt^2+^, and Fe^2+^ metal ions specifically bind to Cys-34 [72]. Ag^+^ has a measured dissociation constant on 1 × 10^−5^ M for its binding to Cys-34 [77].

### 5.3. The Multi-Metal Binding Sites A and B (MBS-A, -B)

The MBS-A is present at the interface of domains I and II [6]. The MBS-A and MBS-B are also known as primary and secondary cadmium binding sites as Cd^2+^ was the first metal ion associated with these sites in NMR studies [78,79]. Site-directed mutagenesis studies show that His-67 present in domain I is crucial for Cd^2+^ binding for MBS-A [79]. The dissociation constant for Cd^2+^ binding at MBS-A and -B is 5.0 × 10^−6^ M [79]. 

X-ray crystallographic studies of apo and metal bound HSA and site-directed mutagenesis analysis have identified Asn-99 present in domain I, His-247, and Asp-249 in domain II as primary residues that coordinate with Zn^2+^ in MBS-A [79,80]. The dissociation constant for Zn^2+^ binding is 2 × 10^−5^ M [80]. It is now well documented that Zn^2+^ ion binds primarily at MBS-A to induce cooperative allostery [72,80]. FA1, FA2, and FA7 binding sites surround the MBS-A, suggesting that the FA loading significantly influences the metal ion affinity to MBS-A [6,72]. The MBS-A additionally binds to Cu^2+^ and Ni^2+^. The multi-metal binding site B (MBS-B) is the fourth and final metal-binding site [6,79,80]. This site primarily associates with Cd^2+^ ions [72]. The amino acids involved in the Cd^2+^ binding at MBS-B are unknown. This site might have a higher degree of flexibility, resulting in multiple conformations; hence, it has not been characterized with confidence by X-ray crystallography or other structural methods. Recently, Co^2+^ ions were also shown to be associated with MSB-B [81].

The toxic effects of metals ions can be detrimental to cell growth, however, some metal ions are required for efficient cell growth as they serve as co-factors for several enzymes involved in critical biological pathways [11]. Albumin, with its ability to interact with various metal ions, can play a dual role in both scenarios. Some metal ions such as vanadium (bound to the drug binding site 1) and selenium (attached to disulfide bonds) are essential for cell growth under in vivo and in vitro conditions [82,83,84]. Albumin coordinates with these trace elements and ensures their transport for optimal cell growth and survival. 

## 6. Antioxidant Features of HSA

The antioxidant activity of HSA is primarily attributed to the redox properties of the four main metal-binding sites [85]. Free Cu^+^, Fe^2+^, and other metal ions react with oxygen to generate ROS [86]. Moreover, they can also interact with H_2_O_2_ to generate harmful hydroxyl radicals [86]. On the other hand, albumin binding to the metal ions limits the ability of these ions to participate in the ROS generation [85]. 

The industrial-scale eukaryotic cell cultures performed in bioreactors contain dissolved oxygen and free metals such as iron, copper, cobalt, and nickel, generating ROS that degrades the cell membranes [11,87]. The inclusion of BSA or HSA leads to lower ROS stress, enabling healthy cell cultures. On the other hand, the binding of albumins to these metals, especially copper, zinc, vanadium, and selenium, facilitates the uptake of these metals by cells, stimulating culture growth and considerably improving recombinant protein production in eukaryotic cells [11].

## 7. Pyridoxal and Riboflavin Interactions

Amino acids are the critical components of cell culture media. Any cell culture medium composition comprises essential amino acids that are required by the cells for efficient translation of the proteins ensuring optimal cell growth, survival, and cell division. Pyridoxal and its derivative pyridoxal 5′phosphate (PLP) react with free amino acids, especially lysine and arginine, to form a Schiff base [88]. These Schiff bases are highly unstable. When exposed to metal ions, they lead to amino acid degradation prohibiting cell growth under in vitro culture conditions. The free pyridoxal moieties are sequestered by binding to albumin [89]. PLP forms a Schiff base with the Lys-190 of HSA [90]. The HSA stabilizes PLP by preventing its degradation [89,91]. It removes the free PLP contaminants from the cell culture media barring PLP–amino acid complex formation.

Riboflavin is another factor that can react with free amino acids to degrade them [92]. It acts as a photosensitizer and oxidizes free amino acids such as tryptophan that are present in the cell culture media. The photoproduct of the riboflavin–amino acid complex is known as lumichrome [92]. This lumichrome formation leads to rapid degradation of the riboflavin-bound amino acids [92,93]. Albumin stabilizes the photoreactive riboflavin. It donates an electron to riboflavin, creating a reduced inactive adduct [94,95]. By doing so, albumin titrates free riboflavins in the cell culture media, eliminating amino acid degradation.

## 8. Chemical Modifications of HSA

Albumins undergo several post-translational modifications that influence the ligand binding and other activity of the proteins [96]. These chemical modifications are acetylation, glycosylation, glycation, nitrosylation, oxidation, carbonylation, phosphorylation, and chlorination [6]. Here, we only discuss essential modifications that influence albumin’s function as an additive in cell culture. Albumin glycation occurs when the amino group of a basic residue forms a Schiff base with a sugar carbonyl group [97,98]. Arg410 and Lys 525 are glycation hotspots in HSA [98]. Upon glycation, albumin shows a significant change in protein conformation caused by a loss in secondary and tertiary structures [99]. Glycation triggers modification of critical residues such as His and Trp, as observed by the loss of intrinsic fluorescence of the protein [100,101]. These modifications also impair the antioxidant properties of albumin [6]. It has been shown that as little as one glycyl group attached to albumin can cause toxicity [6]. Thus, the albumin purification process from plasma or recombinant protein from heterologous sources should avoid albumin glycation.

The Cys34 is another chemical modification hotspot [102,103]. S-nitrosylation of Cys34 alters its metal-binding properties [96]. Cys34 oxidation attributes to the significant antioxidant activity of albumins [96]. Additionally, methionine residues also undergo oxidation to scavenge the reactive oxygen species [104]. Met87, Met123, Met329, Met446, and Met548 are primary residues that act as metal chelators to neutralize the ROS [56]. Together, Met and Cys residues account for 40–80% of the antioxidant activity of HSA [56,85].

## 9. Ligand Interactions

The ligand-binding sites are the primary basis of HSA-based cargo delivery [9,105]. High-resolution structures of HSA with therapeutic compounds alongside biochemical and biophysical studies have characterized three ligand binding sites located in the IIA, IIIA, and IB subdomains (Figure 1). The IIA and IIIA subdomain binding sites are commonly referred to as ‘Sudlow sites’ 1 and 2, respectively [68]. Carter et al. demonstrated the presence of the third major drug-binding region of HSA in the subdomain IB [106,107]. 

The drug-binding site 1 (Sudlow site 1) is present in subdomain IIA. The site is predominantly apolar, with a couple of groups of polar residues. One group of polar residues is present at the very bottom of the site and comprises residues Tyr150, His242, and Arg257 [9]. The second group is located at the opening of the binding pocket and contains residues Lys195, Lys199, Arg218, and Arg222 [9]. The abundance of basic residues defines the ligand-binding specificity of this site. The Sudlow site 1 accepts warfarin, phenylbutazone, amantadine, azapropazone, azidothymidine, indomethacin, iodipamide, oxyphenbutazone, 2′indole sulfate, and 3′diflunisal [9]. 

The subdomain IIIA harbors the drug-binding site 2 (Sudlow site 2) [9]. This ligand-binding pocket is predominantly hydrophobic, with characteristic electrostatic features. Polar residues present at one side of the binding pocket entrance [9]. Arg410, Ser489, and Lys414 are critical residues within this site that interact with associated ligands. Several drug molecules such as ibuprofen, digitoxin, benzodiazepine, halothane, propofol, and non-steroid anti-inflammatory drugs are shown to bind specifically to this drug site 2 [9]. 

A third drug binding site, identified in subdomain IB, has been shown to accommodate lidocaine, bilirubin, warfarin, myristic acid, naproxen, indomethacin, and heme iophenoxic acid [106]. This site offers more affinity towards endogenous ligands and heterocyclic compounds. Tyr138, Tyr161, Arg141, and Lys190 are the critical residues involved in ligand binding site [108]. 

The ligand-binding affinity of all these sites is affected by the conformational changes that occurred due to FA binding [6]. The FAs association with the albumin has shown to increase the binding affinity of ligands such as warfarin to the Sudlow site1 while decreasing the binding affinity of diazepam to the Sudlow site 2 [96]. The Sudlow sites 1 and 2, and third ligand binding site share the amino acid composition with the FA binding hotspots [6]. The FA7-binding site overlaps with Sudlow site 1, and the FA3- and FA4-binding sites overlap with Sudlow site 2 [6]. Allosteric changes resulting in FA binding might affect the ligand interacting side chains of the amino acids lining the binding pocket [6,57,58]. 

The HSA sequesters and transports free heme by interacting with the third drug binding site in the subdomain IB [6]. Heme under physiological conditions serves as a prosthetic group for heme-binding proteins essential for the growth and division of cells. Higher levels of heme can cause generation of ROS, resulting in oxidative stress [109]. Heme also causes the oxidation of high- and low-density lipoproteins [110]. HSA titrates the surplus heme and channels it into the heme degradation pathways, thus helping in cell survival [111]. Similarly, the HSA–bilirubin complex prevents cell death from bilirubin toxicity in culture conditions [112].

## 10. Protein Interactions

Various protein interactions facilitate albumin uptake from the extracellular environment to inside the cell. Specialized plasma membrane surface proteins are involved in these processes. These membrane proteins use clathrin-dependent or dynamin-dependent endocytosis processes. Some of the common membrane-bound albumin-binding proteins are glycoprotein 60 (gp60)/albondin, gp18, gp30, apolipoprotein B-100, IgG receptor FcRn large subunit p51, alpha-2-HS-glycoprotein, apolipoprotein A-I, fibronectin type III, alpha-1-acid glycoprotein 1, antithrombin-III, fibrinogen alpha chain, vascular endothelial growth factor A, and SPARC protein (secreted protein, acidic, and rich in cysteine) [7,113,114,115,116]. More than 50% of albumin is absorbed from the blood capillarylumen by albondin interactions [117]. SPARC protein binds to albumin in a similar fashion to albondin and is shown to enhance drug-bound albumin accumulation within tumorous tissues [116]. The most studied albumin interaction is the FcRn–albumin complex. The FcRn binding site is present in the C-terminal of the domain III of albumin. FcRn binds to albumin in a pH-dependent manner. Site-directed mutation studies show that three conserved histidine residues (H646, H510, and H535) on domain III of HSA play a critical role in FcRn binding. The FcRn regulates the half-life of the bound albumin to about three weeks by protecting albumin from intracellular degradation [118]. Recently, recombinant albumin variants with altered FcRn binding kinetics resulted in an extended albumin half-life [119,120,121]. These protein interactions facilitate albumin trafficking inside the cells in a culture environment. This results in the import of albumin-bound ligands such as metals and FAs essential for optimal cell growth and survival [11].

## 11. Protective Role of Albumin against Physical Damage in Cell Culture

Eukaryotic cells, especially mammalian cells, are susceptible to physical stress in a bioreactor environment [122,123,124,125]. Albumin protects the eukaryotic cell damage in sparged and airlift type bioreactors [126]. Evidence shows that minimal usage (1 g/L) of albumin can significantly reduce cell lysis in mammalian cell cultures in pilot airlift bioreactors or bubble-free membrane aerated bioreactors [127]. Albumins are also used along with other additives such as anti-foaming agents and pluronic acid as a shear protectant for optimal results [128]. It is now widely believed that albumins might interfere with the cell culture’s physiochemical properties to prevent physical cell damage [128]. It is, however, largely unclear how additives, especially proteins such as albumin, prevent acute lethal cell damage in a bioreactor environment [129,130].

## 12. Role of Albumins in Bioprocess Development

Albumins have now wholly replaced the usage of serum and its derivatives in bioprocess development on an industrial scale. This is possible only with the availability of desirable quantities of highly purified recombinant albumins expressed and purified from heterologous hosts [11]. It have been shown to maintain cell stability in bioreactors for producing interleukin-2 and other therapeutic proteins [127]. Albumins are used in antibody production in hybridoma cell culture media [131]. Albumins support the growth of the immortal cell lines in the culture medium, working as a physical shear protectant [11,132]. HSA is also used in stem cell culture applications to promote the highly reproducible differentiation of human embryonic stem cells [133]. HSA also promotes growth of mouse embryo culture [134]. rHSA is highly desirable for these purposes as it meets stringent regulatory requirements for clinical applications [23]. Albumin is used for the development of serum-free media for fibroblast culture [135,136]. Not all albumin has the same efficacy in the cell culture media. The purity of the protein and the composition of albumin-associated ligands are the key factors that govern the protein’s role as an additive in culture media [11,137]. The ligands associated with the purified proteins heavily depend on the protein source (serum or recombinant) and the purification process [138]. This is the main reason for batch-to-batch variation in the cell culture with albumin as an additive [138,139]. The addition of stabilizers, such as octanoic acid, with rHSA compositions has been shown to have profound adverse effects in specific cell culture systems [140]. Moreover, different post-translational modifications, including glycation and oxidation in HSA from recombinant or serum origins, produce significant batch-to-batch variability [141]. It is generally advised to stick with one vendor and albumin manufacturer while optimizing a cell culture process.

Albumins are substituted by other synthetic or natural alternatives in modern-day cell culture media [142]. Hydrolysates, especially plant protein hydrolysates, are successfully used to substitute albumins in embryo culture media [143]. Hyaluronic acid is also used to replace albumin in the human embryo transfer medium [144]. Polyvinyl alcohol combined with amino acids is used to substitute albumins in culture medium for mouse pre-implantation embryos. Another popular approach is the protein-free cell culture media [145]. Proprietary formulations such as MEM and RPMI-1640 are protein-free. Compared to albumin-containing media, protein-free media can promote superior cell growth, higher protein expression, and facilitate simpler downstream purification for many recombinant proteins [146]. However, these formulations are usually cell-line specific and need to be verified or optimized for any given culture. 

## 13. Conclusions

The inherent properties of the human serum albumin make it highly desirable for biotechnological applications. It is commonly used in cell culture applications as a replacement for traditionally used serum. It is a crucial transporter of biologically active components essential for the growth and survival of eukaryotic cells. With overgrowing applications in hybridoma culture media, stem cell culture media, and tissue engineering media, albumins are becoming more relevant to modern-day bioprocess development. High-resolution structures of HSA have identified critical residues for ligand binding and transport, which are essential for the growth and development of cells. This information can now be exploited by the modern-day recombinant DNA technology to design specific HSA variants in array-based experiments to test custom ligand transport, enhanced metal binding, or potent ROS scavenging activities, both in vivo and in vitro, in a cost-effective manner.
